# The Effect of Glucagon-Like Peptide-1 (GLP-1) Receptor Agonists on the Lipid Profile of Diabetic Patients Using Statins: A Retrospective Cohort Study in the Diabetic Center of King Salman Bin Abdulaziz Hospital, Saudi Arabia

**DOI:** 10.7759/cureus.65521

**Published:** 2024-07-27

**Authors:** Odai M Albahli, Saqib Ali, Fahad Alblaihi, Abdulaziz A Aljaman

**Affiliations:** 1 Family Medicine, Kingdom of Saudi Arabia Ministry of Health, Riyadh, SAU; 2 Endocrinology and Diabetes, King Salman Hospital, Riyadh, SAU

**Keywords:** statins, diabetes mellitus, dyslipidemia, dulaglutide, glp-1ra

## Abstract

Background

Dyslipidemia is a common complication of type 2 diabetes mellitus (T2DM), which is the leading cause of morbidity and mortality in developed countries, including Saudi Arabia. Injectable glucagon-like peptide-1 receptor agonists (GLP-1RA) are recent therapies found to be effective in treating dyslipidemia in diabetic patients by acting similarly to the body's internal glucagon peptide hormone, regulating blood sugar levels and reducing triglycerides and low-density lipoprotein (LDL) levels.

Aim of the study

This retrospective cohort study aimed to investigate the effect of GLP-1RA on the lipid profile of dyslipidemia diabetic patients who were not controlled using statins.

Methods

Data were collected from the medical records of male and female diabetic patients with dyslipidemia (uncontrolled by statins) who were administered to the diabetic center of King Salman Bin Abdulaziz Hospital in Riyadh, Saudi Arabia, and received GLP-1RA (dulaglutide) from June 2023 to August 2023. The primary endpoint was the change in triglycerides and LDL-C levels after three months of using dulaglutide, and the secondary endpoints included the change in body weight, BMI, and HbA1c%. Descriptive analysis was conducted to present numerical and categorical data. The Wilcoxon signed-rank test was used to compare numerical data before and after using dulaglutide. Ethical considerations were taken into account by ensuring anonymous data collection and obtaining IRB approval before data collection.

Results

The study included 102 patients with a median (interquartile range (IQR)) age of 59 (14) years. Females constituted 55.2% of the population. Obesity (96.1%), hypertension (71.6%), and retinopathy (13.7%) were the most commonly reported comorbidities. The study showed a significant reduction in body weight, BMI, HbA1c, hemoglobin, mean corpuscular volume (MCV), serum LDL-C, and triglyceride concentrations after three months of using dulaglutide (p<0.001).

Conclusion

Our study results confirm the positive effect of the GLP-1RA (dulaglutide) on the lipid profile of diabetic patients with dyslipidemia uncontrolled by statins.

## Introduction

Type 2 diabetes mellitus (T2DM) is a chronic disease affecting 387 million people worldwide, and it is estimated to reach 600 million by 2035 [1]. Locally, in the Kingdom of Saudi Arabia, the incidence of T2DM is 32.8%, and it is also predicted to reach 45.36% by 2030 [2]. In fact, the World Health Organization (WHO) ranked Saudi Arabia as the second country in the Middle East with a high prevalence of T2DM [3]. T2DM is defined as a metabolic syndrome with deficient insulin signaling in adipose tissues and skeletal muscles, resulting in exhaustion of the beta cells in the pancreatic islets and chronic hyperglycemia [4,5].

Several chronic conditions are associated with uncontrolled T2DM, such as dyslipidemia, cardiovascular diseases, myocardial and cerebral infarctions, neuropathy, retinopathy, and nephropathy [6]. As a common metabolic comorbidity of T2DM, dyslipidemia (abnormal plasma lipids and lipoprotein levels) accounts for high mortality rates in developed countries, including Saudi Arabia [7,8].

T2DM management involves various interventions, including lifestyle changes and medications such as sulfonylureas, thiazolidinediones, glucosidase inhibitors, dipeptidyl peptidase 4 (DPP-4) inhibitors, metformin, and insulin therapies, all of which contribute to substantial improvements in blood glucose [4]. However, focusing on treatment strategies that address T2DM comorbidities is found to be more efficient [9]. Glucagon-like peptide-1 receptor agonists (GLP-1RA) mimic the pancreatic glucagon peptide hormone, regulating blood sugar levels and activating insulin production [4]. Moreover, incorporating GLP-1RA into the treatment regimen of diabetic patients could be useful in cases such as metformin intolerance or contraindication, patients with sustained hemoglobin A1c greater than 1.5% above the target range over three months, and patients with chronic kidney diseases or heart failure [10].

GLP-1RAs were found to be effective in reducing total cholesterol, low-density lipoprotein (LDL), and triglyceride (TG) levels by blocking their absorption from the small intestine, inhibiting hepatic production, and inactivating very low-density lipoprotein (VLDL) production. Thus, they lead to effective weight loss and improvements in dyslipidemia. However, there are several knowledge gaps regarding the efficacy of GLP-1RAs in this regard, including the underlying pathophysiological mechanism and the relation between statins and GLP-1RA in LDL-C reduction [11]. Therefore, this study aimed to determine the effect of three months of dulaglutide treatment on the lipid profile of T2DM patients with dyslipidemia uncontrolled by statins.

## Materials and methods

Study design and participants

This observational retrospective cohort study was conducted at the diabetic center of King Salman Bin Abdulaziz Hospital in Riyadh, Saudi Arabia. The study included both male and female patients aged over 18 with T2DM and dyslipidemia uncontrolled by statins (atorvastatin), even at the maximum tolerable dose. Data were collected from the medical records of eligible subjects who initiated GLP-1RA therapy (dulaglutide) in June 2023, with follow-up data available for three months (until August 2023). Patients with incomplete medical records were excluded from the study.

Data collection

At baseline (before initiation of dulaglutide therapy), demographic data (age, gender, body weight, height, body mass index (BMI), nationality, and educational level), clinical data (comorbidities, diabetic complications, and antidiabetic medications), and laboratory data (HbA1c, hemoglobin level, mean corpuscular volume (MCV) level, serum TGs, and LDL) were collected. After three months of follow-up, data on body weight, height, BMI, and laboratory data were collected.

Statistical analysis

Statistical analysis was conducted using the computer program IBM SPSS Statistics for Windows, Version 26 (Released 2019; IBM Corp., Armonk, New York). Baseline data were reported using descriptive statistics. The normality of distribution for continuous variables was analyzed using the Shapiro-Wilk test. The mean and standard deviation (SD) were calculated for parametric data, while the median and IQR were calculated for nonparametric data. Categorical variables were expressed as absolute numbers and valid percentages. Wilcoxon signed-rank test was used to compare numerical data collected after three months of follow-up to baseline values. Statistical significance was considered when the p-value was less than 0.05.

## Results

The analysis included data from the medical records of 102 T2DM patients with dyslipidemia not controlled by statins who were prescribed dulaglutide 1.5 mg for at least three months. The mean (SD) age of the included patients was 60.4 (8.8) years. More than half (37, 55.2%) were females, and the vast majority (52, 92.9%) were of Saudi nationality. The most common comorbidities among the studied patients were obesity (98, 96.1%), hypertension (73, 71.6%), and retinopathy (14, 13.7%) (Table 1).

**Table 1:** Baseline demographic characteristics of the patients (n=102) SD: standard deviation; IQR: interquartile range

Parameters	Category	Number	Percentage
Age (years)	Mean (SD)	60.4 (8.8)	-
	Median (IQR)	59 (14)	-
Gender (n=67)	Female	37	55.2
	Male	30	29.4
Nationality (n=56)	Saudi	52	92.9
	Non-Saudi	4	7.1
Educational level (n=47)	Undergraduate	19	40.4
	Graduate	28	59.6
Comorbidities (n=102)	Obesity	98	96.1
	Hypertension	73	71.6
	Retinopathy	14	13.7
	Myocardial infarction	10	9.8
	Cerebral infarction	5	4.9
	Nephropathy	3	2.9
	Others	3	3

All included patients were prescribed dulaglutide 1.5 mg/0.5 mL for at least three months. In addition to dulaglutide, the most commonly used antidiabetic medications were metformin (92.2%), empagliflozin (15.7%), and sitagliptin (8.8%). After three months of using dulaglutide, the data showed a statistically significant reduction in all assessed parameters.

The median (IQR) body weight of the studied patients at baseline, 91 (15.5) kg, significantly decreased (p<0.001) to 87.3 (13.8) by 5 (3.75) kg after three months of using dulaglutide with median (IQR) decrease value of 5 (3.75) kg and a median (IQR) percent reduction of 5.6% (2.7). In addition, the median (IQR) BMI at baseline, 33.8 (5.2) kg/m², significantly decreased (p<0.001) to 31.1 (4.7) kg/m² after three months. The median (IQR) reduction in BMI was 3 (2) kg/m², with a median (IQR) percent reduction of 8.7% (4.3).

The same as body weight and BMI, the median (IQR) HbA1c at baseline, 8.9% (0.5), significantly decreased (p<0.001) to 8.2% (0.7) after three months of follow-up. The median (IQR) change in HbA1c was -0.9% (0.7), with a median (IQR) percent reduction of 10.1% (7.8).

Regarding the lipid profile, the median (IQR) serum TG level at baseline was 184 (80) mg/dL, which significantly decreased (p<0.001) to 137 (79.3) mg/dL after three months of follow-up with a median (IQR) reduction of 20 (61.8) mg/dL and a median (IQR) percent reduction of 20.2% (31.5).

In addition to the reduction in TG levels, the median (IQR) LDL-C at baseline, 135.5 (74) mg/dL, was significantly decreased (p<0.001) to 92.5 (56) mg/dL after three months of follow-up. The median (IQR) reduction in LDL-C was -25.5 (56) mg/dL, with a median (IQR) percent reduction of 22.76% (40.5).

**Figure 1:**
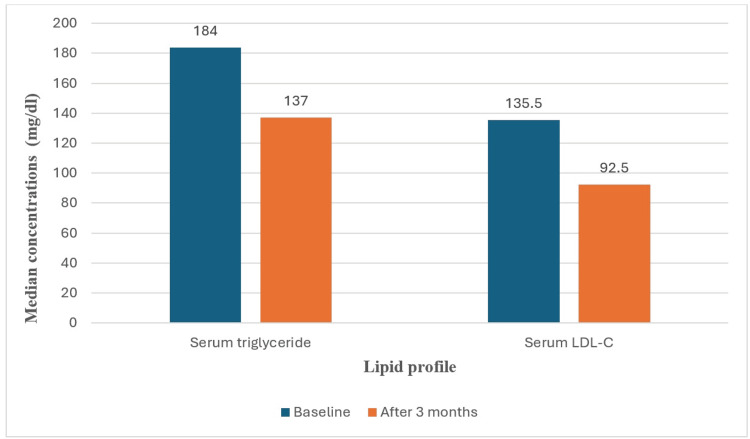
Change in the lipid profile after three months of using dulaglutide LDL-C: low-density lipoprotein cholesterol

Regarding hemoglobin level, there was a statistically significant decrease (p<0.001) in the median (IQR) value at baseline, 13 (3) g/dL, to reach 12 (4) g/dL after three months. The median (IQR) change in hemoglobin was -0.5 (1.5) g/dL, with a median (IQR) percent reduction of 3.8%. The median (IQR) MCV at baseline was 90 (5) fL, which significantly decreased (p<0.001) to 86 (11) fL after three months with a median (IQR) change of -3 (9) fL and a median (IQR) percent reduction of 3.2% (11). More details are provided in Table 2 and Figure 1.

**Table 2:** Laboratory variables at the initiation of dulaglutide treatment and after the three-month follow-up period IQR: interquartile range; BMI: body mass index; HbA1c: hemoglobin A1C; MCV: mean corpuscular volume; LDL-C: low-density lipoprotein cholesterol

Variable	Baseline Median (IQR)	Median (IQR) After the Three-Month Follow-Up	Change Median (IQR)	% Change Median (IQR)	p-value
Weight (kg)	91 (15.5)	87.3 (13.8)	-5 (3.75)	-5.6 (2.7)	<0.001
BMI (kg/m^2^)	33.8 (5.2)	31.1 (4.7)	-3 (2)	-8.7 (4.3)	<0.001
HbA1c (%)	8.9 (0.5)	8.2 (0.7)	-0.9 (0.7)	-10.1 (7.8)	<0.001
Hemoglobin (g/dL)	13 (3)	12 (4)	-0.5 (1.5)	-3.8 (12)	<0.001
MCV (fL)	90 (5)	86 (11)	-3 (9)	-3.2 (11)	<0.001
Serum triglyceride (mg/dL)	184 (80)	137 (79.3)	-20 (61.8)	-20.2 (31.5)	<0.001
LDL-C (mg/dL)	135.5 (74)	92.5 (56)	-25.5 (56)	-22.76 (40.5)	<0.001

## Discussion

During the last decade, significant efforts have been made to improve the lipid profile, body weight, and blood sugar levels in T2DM patients. Despite the use of multiple antidiabetic and lipid-lowering therapies, GLP-1RA, such as dulaglutide, is considered a substantial solution that enhances the quality of life of suffering patients [11]. This study could add to the body of knowledge and show possible efficient management of dual metabolic syndromes, diabetes, and dyslipidemia; therapy could decrease inadequate patient adherence to multiple medications.

Several studies have investigated the long-term effect of GLP-1RA on the lipid profile [12-14]. However, this retrospective cohort study aimed to assess the short-term effect of dulaglutide on the lipid profile of dyslipidemia diabetic patients in the diabetic center of King Salman Bin Abdulaziz Hospital in Riyadh, Saudi Arabia.

Despite the short-term duration, the addition of dulaglutide in this study to the dyslipidemia diabetic patients who are already following atorvastatin and antidiabetic medications showed successful improvements in many parameters. In our study, the mean age was 60.4 ± 8.81 years, similar to the previous study, which obtained a mean age of 63.8 years [12]. Clearly, this is the most common mean age of T2DM patients since the literature reports that the mean age at diagnosis of diabetes is 54.60 ± 9.48 years [15].

In accordance with several studies [16-18], our study findings regarding body weight and BMI showed a significant reduction (p<0.001) by 5 kg and 3 kg/m^2^, respectively, quite surprisingly, in only three months of follow-up after the administration of dulaglutide, along with those of Ishigaki et al., which found that the body weight decreased by 1.3 kg and the BMI by 0.5 kg/m^2^ after six months of administration of GLP-1RA treatment [12]. However, this may be a result of the large sample size (932) compared to ours, which can lead to some variations in the results.

Additionally, a similar study conducted in Saudi Arabia showed a significant decrease in BMI and body weight for patients following statins after initiating GLP-1RA [13]. The underlying mechanism is well documented. The GLP-1RA increases satiety, lowers appetite, delays gastric emptying, and reduces intestinal motility by increasing insulin secretions [4].

Regarding glycemic control, using dulaglutide along with other antidiabetic medications revealed a clinically significant reduction (p<0.001) in the HbA1c levels by 0.9% compared to the findings of Ishigaki et al., who found a significant decrease in HbA1c by 0.45% after using dulaglutide after six months of follow-up [12]. This is consistent with Ajabnoor et al.'s findings [13] and opposing Alanazi et al.'s findings [16], which did not find a significant reduction in HbA1c. This is attributed to the reported effect of GLP-1RA on improving beta cell functions, blocking glucagon secretion, and enhancing insulin sensitivity [19].

Furthermore, this study found a significant marked reduction (p<0.001) in the serum TG concentrations by 20 mg/dL and LDL-C by 25.5 mg/dL after following dulaglutide for three months. Similarly, Ishigaki et al. revealed a significant decrease in TG concentrations by 10.8 mg/dL and LDL-C concentrations by 2.2 mg/dL after administration of GLP-1RA for six months [12]. In contrast, a study conducted by Ajabnoor et al. found no association between GLP-1RA therapy and lipid profile changes [13]. However, our study findings align with those of other studies [20,21].

Another potential finding is a significant reduction in hemoglobin levels and MCV; in other words, dulaglutide could be associated with anemia in dyslipidemia diabetic patients as an adverse effect. A similar pattern of results was observed in a case report conducted in Saudi Arabia regarding a 30-year-old male who demonstrated acute hemolytic anemia following semaglutide administration. His anemia improved once he stopped taking semaglutide [22]. In addition, these results tied well with a study that found that patients who received SGLT2 inhibitors had a lower prevalence of composite anemia compared to those who received GLP-1RA. However, the mechanism of hemoglobin reduction associated with GLP-1RA is still unknown [15]. Nonetheless, we cannot neglect the effect of statins taken by the patients, as Ahn et al. revealed a significant fivefold increase in the risk of iron deficiency anemia following the administration of statins to the patients [23].

Strengths and limitations

The main strength of the study is that it collects real-world data on various metabolic parameters from patients' medical records, which enhances the generalizability of the results. One of the limitations of the current study is that it is a single-center study with a relatively small sample size and a short duration of follow-up. In addition, the retrospective nature of the study could include a risk of selection bias, the lack of a control group, the potential presence of confounding factors, and missing/incomplete data records. We recommended conducting future multicenter comparative studies with a larger sample size to get in-depth information about the safety and efficacy of dulaglutide in improving the lipid profile of patients with T2DM.

## Conclusions

In the current study, using GLP-1RA (dulaglutide) for three months was associated with a significant reduction in body weight, BMI, HbA1c, hemoglobin, MCV, serum LDL-C, and TG levels in T2DM patients with dyslipidemia who were not controlled by statins. Adding dulaglutide to the antidiabetic and antihyperlipidemic treatment regimen could potentially improve the lipid profile, aid in body weight reduction, and consequently enhance the quality of life of these patients. In addition to showing efficient management of dual metabolic syndromes, diabetes, and dyslipidemia, it could decrease inadequate patient adherence to multiple medications.
